# Evidence for salicylic acid signalling and histological changes in the defence response of *Eucalyptus grandis* to *Chrysoporthe austroafricana*

**DOI:** 10.1038/srep45402

**Published:** 2017-03-28

**Authors:** Lizahn Zwart, Dave Kenneth Berger, Lucy Novungayo Moleleki, Nicolaas A. van der Merwe, Alexander A. Myburg, Sanushka Naidoo

**Affiliations:** 1 Department of Genetics, Forestry and Agricultural Biotechnology Institute (FABI), Genomics Research Institute (GRI), University of Pretoria, Pretoria, South Africa; 2 Department of Plant and Soil Sciences, Forestry and Agricultural Biotechnology Institute (FABI), Genomics Research Institute (GRI), University of Pretoria, Pretoria, South Africa; 3 Department of Microbiology and Plant Pathology, Forestry and Agricultural Biotechnology Institute (FABI), University of Pretoria, Pretoria, South Africa

## Abstract

*Eucalyptus* species are cultivated for forestry and are of economic importance. The fungal stem canker pathogen *Chrysoporthe austroafricana* causes disease of varying severity on *E. grandis.* The *Eucalyptus grandis-Chrysoporthe austroafricana* interaction has been established as a model system for studying *Eucalyptus* antifungal defence. Previous studies revealed that the phytohormone salicylic acid (SA) affects the levels of resistance in highly susceptible (ZG14) and moderately resistant (TAG5) clones. The aims of this study were to examine histochemical changes in response to wounding and inoculation as well as host responses at the protein level. The anatomy and histochemical changes induced by wounding and inoculation were similar between the clones, suggesting that anatomical differences do not underlie their different levels of resistance. Tyloses and gum-like substances were present after inoculation and wounding, but cell death occurred only after inoculation. Hyphae of *C. austroafricana* were observed inside dead and living cells, suggesting that the possibility of a hemibiotrophic interaction requires further investigation. Proteomics analysis revealed the possible involvement of proteins associated with cell death, SA signalling and systemic resistance. In combination with previous information, this study forms a basis for future functional characterisation of candidate genes involved in resistance of *E. grandis* to *C. austroafricana*.

The *Eucalyptus grandis* genome^1^ was sequenced recently and this has facilitated omics studies such as the description of the repertoire of R genes in this species^2^ and expression profiling experiments^3^,^4^, providing valuable resources for studying *E. grandis* defence. The pathosystem of *E. grandis* and the stem canker pathogen *Chrysoporthe austroafricana* has been established as a model system for studying antifungal defence in this commercially important tree species^5^. Two *E. grandis* clones have been selected for use in this model system based on their different levels of resistance to *C. austroafricana*^3^,^6^. These clones, which are moderately resistant (TAG5) and highly susceptible (ZG14) can be compared to identify factors that increase resistance to *C. austroafricana* infection in *E. grandis.* In this model pathosystem, artificial inoculation involves wounding of the bark in order to place fungal mycelium on living xylem tissue. This simulates natural infection, which is thought to occur through wounds. Inoculation is therefore expected to induce responses to wounding and fungal infection.

In a study by Naidoo *et al*.^5^, SA and jasmonic acid (JA) signalling appeared to be antagonistic based on the expression of hormone marker genes. In addition, the SA marker gene PR2 was induced earlier in TAG5 than ZG14 after inoculation, and treatment of ZG14 with SA restored its level of resistance to that of TAG5. This was a surprising response to a suspected necrotroph, which would be expected to proliferate more efficiently during a SA-mediated response.

A subsequent investigation^3^ of the transcriptomic changes in these two clones at the site of inoculation revealed the importance of SA and other phytohormones in the interaction, with the possibility of a delayed response in ZG14, as well as the involvement of genes associated with systemic resistance only in TAG5. To understand the role of the pathogen in the interaction, the *C. austroafricana* draft genome was used to perform a dual RNA-Seq analysis^7^. Several putative virulence genes were identified, and the results suggested possible manipulation of SA and gibberellic acid (GA) signalling as well as plant cell wall degradation by the pathogen. In the same study, light microscopy revealed that the pathogen occurred throughout the stem tissue, appeared to spread by penetrating cell wall pits, and that lesion development coincided with pathogen spread.

It is possible that pre-existing anatomical barriers affect pathogen spread. Histological changes induced by wounding and inoculation could contribute to the level of host resistance, and it is not known whether *C. austroafricana* occurs inside living or dead cells. In this study, light microscopy was used to investigate these three aspects of the interaction in TAG5 and ZG14. In addition, quantitative proteomics was used to investigate the role of phytohormone signalling in the antifungal defence response of the *E. grandis* clone TAG5. The experimental design allowed the identification of responses to wounding and inoculation, which can be useful for identifying infection-specific processes. The purpose of this study was to contribute to the current understanding of the factors affecting resistance to *C. austroafricana* in *E. grandis*, with a particular focus on the role of SA and the identification of candidate defence genes for functional characterisation.

## Results

### Host anatomical and histochemical responses

While the location and spread of *C. austroafricana* during infection of *E. grandis* have been described^7^, the responses of *E. grandis* to infection with *C. austroafricana* have not been studied at the microscopic level. This information could provide clues about the physical changes in the host during wounding and infection, some of which could contribute to resistance, and about pathogen lifestyle.

#### Inoculation trial and macroscopic changes after wounding and inoculation

Lesion formation similar to previous reports^3^,^5^,^7^ was observed in all inoculated plants. After 42 days, the wounded plants had formed new callus tissue that occluded all or most of the original wound (Supplementary Figure S1A). Callus was absent from the inoculated plants. Wilting and death occurred in some of the inoculated plants by 42 dpi^7^.

#### Histochemical changes during the interaction with *Chrysoporthe*

**Figure 1:**
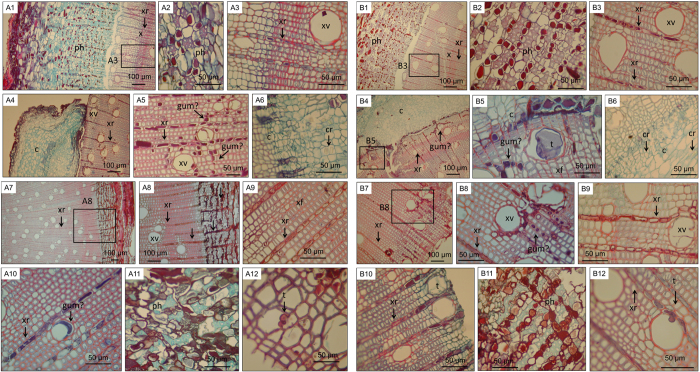
Cross-sections of TAG5 (**A**) and ZG14 (**B**) stems stained with safranin and fast green. The samples were unwounded (A1–A3,B1–B3), or collected at 42 dpw (A4–A6,B4–B6), 3 dpi (A10,B10), 7 dpi (A7–A8), 14 dpi (A9,A11,A13,B11,B12), 21 dpi (B7–B8), and 42 dpi (B9). ph: phloem, x: xylem, phr: phloem ray, xr: xylem ray, c: callus, cr: crystal, xv: xylem vessel, xf: xylem fiber, t: tylose. Blocks indicate areas also shown at higher magnification.

Host responses of *E. grandis* during wounding and inoculation were investigated by viewing tangential longitudinal and cross sections of tissue from the site of wounding or inoculation, or tissue approximately 30 cm above the base of the stem in unwounded controls. The results presented here are based on all of these observations and the images shown are meant to illustrate the most pertinent findings. The anatomy of unwounded plants is shown in Fig. 1(A1–A3. Almost no tyloses or dark-staining areas were present in xylem vessel and fibre lumina. Staining with Lugol’s solution revealed that starch granules were particularly abundant in the xylem ray parenchyma (Supplementary Figure S1B). Diffuse porous wood was observed, with uniseriate rays and pith. Pits were present in xylem fibre and vessel walls. Apart from bark thickness, no differences in the anatomy of the two clones were apparent.

After artificial wounding, dark-staining substances and darker staining of xylem ray parenchyma cells (Fig. 1A4) were observed around the wound site. Tyloses occurred within xylem vessel and fibre lumina (Fig. 1B4). New growth in the form of callus was observed near the cambial zone. These callus cells initially had blue-staining cellulose-rich walls and were uniform in size and appearance. At later time points, crystals and lignin-rich red staining of cell walls were observed in the callus (Fig. 1A4). The wounding responses were similar in TAG5 and ZG14 at this resolution.

Inoculated samples exhibited anatomical changes similar to those seen in wounded controls (Fig. 1A10). Dark-staining substances were observed (Fig. 1A10) and tyloses were present in xylem vessels of both clones (Fig. 1A12). The main difference between inoculated and wounded plants was the apparent damage to living cells in the bark and xylem consisting of discolouration around the cell wall, loss of cell contents, disruption of cell morphology and tissue disorganisation after inoculation (Fig. 1A7–A9). This was also apparent in longitudinal sections (not shown). This damage coincided approximately with the location of the visible stem lesion; sections containing tissue that did not contain macroscopically visible lesions did not contain these damaged cells, while tissue from within the visibly affected area did. In contrast, wounded samples were healthy in appearance and any damage was limited to a small area immediately next to the wound. Very little or no new growth was seen in sections of inoculated samples. The initial observations detailed here show that there are no drastic differences in the histological responses to infection between the two clones. However, since the rate of lesion development differs between these clones^3^,^5^,^7^ and cell damage coincides with the appearance of the lesion, it is possible that the rate of cell damage is different in these clones.

#### Vital staining of *E. grandis* stems infected with *C. austroafricana*

*Chrysoporthe austroafricana* is suspected to be a necrotrophic pathogen. However, to our knowledge, this has not formally been tested. In a previous study, microscopic examination of *E. grandis* samples inoculated with *C. austroafricana*^7^ showed that hyphae appeared to enter living xylem ray parenchyma cells through cell wall pits. It was not clear whether hyphae were present within these cells, since staining masked the cell contents, or whether these xylem ray parenchyma cells were indeed living. The presence of hyphae within living cells could be indicative of a biotrophic phase in the fungal life cycle^8^. Furthermore, it was necessary to determine whether the damaged cells seen in inoculated samples were indicative of cell death. These possibilities were tested by vital staining of fresh, unfixed tissue from wounded and inoculated TAG5 and ZG14 stems.

**Figure 2:**
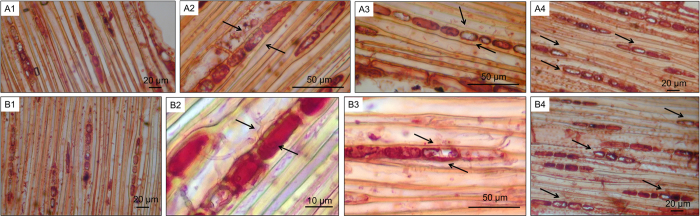
Vital staining of longitudinal sections from TAG5 (**A**) and ZG14 (**B**) stems wounded (A1,B1) or inoculated with *C. austroafricana* (A2–A4,B2–B4). sg: starch granules, xf: xylem fibres, xr: xylem ray. Arrows indicate hyphae (A2–A3,B2–B3) and dead xylem ray parenchyma cells (A4,B4).

Failure of cells to stain does not necessarily imply that they are dead, since sample processing could cause damage and imperfect staining efficiency. Therefore, only stained cells surrounded by other stained cells were considered living, and only unstained cells surrounded by stained cells were considered dead^9^. In both TAG5 and ZG14, hyphae were clearly visible within living cells (Fig. 2A2). Dead cells containing hyphae could also be seen (Fig. 2A3). *Chrysoporthe* did not appear to form specialised haustoria in *E. grandis* cells, but the resolution prohibited unambiguous visualisation of the entire hyphal structure within a cell. In addition, vital staining revealed that the cells exhibiting damage after inoculation are indeed dead (Fig. 2A4) while this was not observed in wounded samples (Fig. 2A1). These results suggest that *C. austroafricana* is not necessarily a necrotroph and that cell death occurs mainly in inoculated samples.

### Quantitative proteomics

#### Protein identification

Quantitative proteomics analysis of the more resistant clone TAG5 was performed to identify processes that could influence its higher level of resistance. From a total of 359 752 spectra (112 153 unique), 31 628 peptides (28 332 unique) and 6 561 proteins were identified (Supplementary Table S1). As expected, most differentially expressed (DE) proteins (Supplementary Figure S2) were present in the Inoculated/Unwounded group (607 up-regulated and 448 down-regulated), followed by Wounded/Unwounded (563 up-regulated and 401 down-regulated) and Inoculated/Wounded (326 up-regulated and 166 down-regulated).

#### Proteomic changes in response to wounding and inoculation

**Figure 3:**
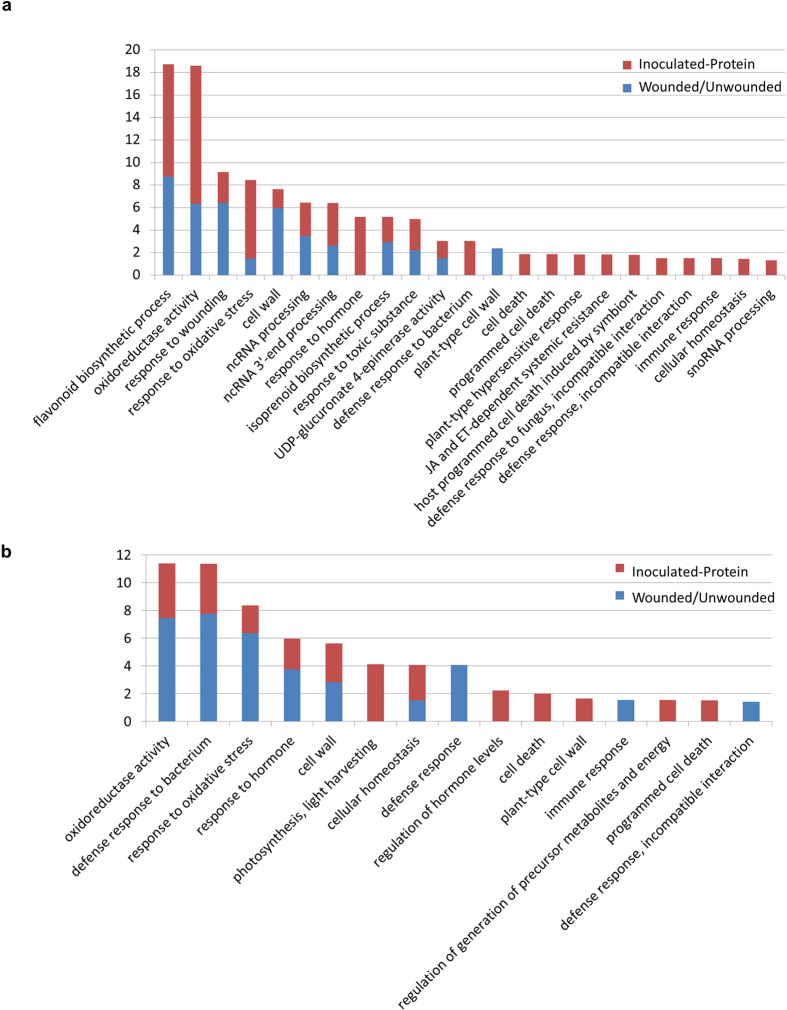
Selected GO terms enriched among the up-regulated (**a**) and down-regulated (**b**) proteins of TAG5. Red bars indicate the differentially abundant proteins in the combined inoculated group (Inoculated-Protein) and blue bars indicate proteins altered in response to wounding (Wounded/Unwounded). The −log2(q-value) is shown on the y-axis and the GO terms on the x-axis.

The experimental design allowed the identification of proteins induced by infection in two ways: subtracting wounding-specific proteins in Wounded/Unwounded from the DE proteins in Inoculated/Unwounded, and comparing the wounded and inoculated samples directly (Inoculated/Wounded). Knowledge about the wounding response facilitates the identification of possible infection-specific proteins and processes that could underlie the observed histological changes in each response. The Wounded/Unwounded and Inoculated/Unwounded groups were compared to identify shared and unique DE proteins. The proteins identified within each group were matched to their TAIR10 identifiers and analysed with BiNGO to identify enriched GO terms within the biological process, cellular component and molecular function categories (Fig. 3).

The overrepresented GO terms that occurred in both the Wounded/Unwounded and Inoculated/Unwounded groups related mainly to the flavonoid and Shikimate pathways among the up-regulated proteins. Other terms suggest the involvement of oxylipins such as jasmonate, as well as redox and antioxidant activity. Terms related to photosynthesis and energy metabolism were shared among the down-regulated proteins, as were several defence and stress associated terms (Fig. 3).

Certain GO terms were unique to each of these two groups. Most cell death related terms were unique to Inoculated/Unwounded, with some terms common to both up- and down-regulated datasets. The terms “host programmed cell death induced by symbiont” and “plant-type hypersensitive response” occurred within the up-regulated dataset. Several terms associated with responses to biotic stimuli were unique to Inoculated/Unwounded, including “defence response to fungus, incompatible interaction” and “immune response”. The term “jasmonic acid and ethylene-dependent systemic resistance, ethylene mediated signaling pathway” is in accordance with previous observations that systemic responses to *C. austroafricana* infection could occur in *E. grandis*^3^.

GO terms unique to the up-regulated subset of Wounded/Unwounded indicated that responses to biotic stimuli and ATPase-mediated transmembrane transport may be increased. Several immune system terms such as “defence response, incompatible interaction” and “innate immune response” were enriched within the down-regulated dataset of Wounded/Unwounded.

The proteins that are only DE in response to infection can also be identified by comparing the inoculated and wounded samples (Inoculated/Wounded). The proteins unique to Inoculated/Unwounded and not DE in Wounded/Unwounded were compared to those identified in the Inoculated/Wounded group. While there was a substantial degree of overlap in the proteins and GO terms identified, a few were unique to each comparison. Upon closer examination, many of the unique GO terms appeared to be involved in similar processes such as secondary metabolite production and immune system processes. A number of GO terms within the Inoculated/Wounded group were also shared between the Wounded/Unwounded or Inoculated/Unwounded groups (Fig. 3). The terms relating to cell death had no similar counterpart in Inoculated/Wounded, but the overall patterns in known defence-related GO terms were otherwise very similar. The proteins identified with these two approaches were combined into a single set of proteins DE in response to inoculation (Inoculated-Protein).

MapMan was subsequently used to identify patterns in specific pathways and processes among the DE proteins. Several flavonoid-related terms were enriched among the up-regulated proteins of all three groups. Visualisation in MapMan showed that the DE genes and proteins occurred in similar parts of the “Flavonoid” pathway in all groups. A cinnamoyl-CoA reductase gene (CCR1, Eucgr.C01240) was up-regulated only in the inoculated groups. This enzyme catalyses the first committed step of lignin biosynthesis. Many other flavonoid pathway proteins were present in all of the comparisons, including phenylalanine ammonia lyase (PAL), cinnamyl alcohol dehydrogenase (CAD), 4-coumarate:CoA ligase (4CL), dihydroflavonol-4-reductase (DFR) and leucoanthocyanidin reductase (LAR). The latter two enzymes are involved in catechin biosynthesis.

The “Biotic stress” overview pathway in MapMan showed that genes involved in similar processes were DE in all datasets. More PR proteins were up-regulated in response to inoculation than wounding. Hormone signalling genes presented a complex pattern of expression, with up- and down-regulated genes and proteins present in almost all categories in each dataset.

The “receptor-like kinases” pathway showed that at a LRR receptor orthologue of a gene with known involvement in defence was up-regulated in the proteome of inoculated samples (AT1G47890, Eucgr.F00700). The “Transcription” pathway revealed that several MYB and MYB-related transcription factors are DE after inoculation, but not wounding. One of these putative MYB TFs (Eucgr.J02939) is orthologous to an *Arabidopsis* protein that is involved in the response to auxin, GA, JA and ET (AT3G16350). This TF was up-regulated in the inoculated proteome and down-regulated in both transcriptomes. Lee *et al*.^10^ identified a MYB transcription factor that was expressed at higher levels in susceptible compared to resistant rice after inoculation with the hemibiotroph *Magnaporthe grisea*. The MYB was expressed at higher levels in the absence of SA. The GO term enrichment results were used to identify candidate genes that could be involved in the higher level of resistance seen in TAG5.

#### Specific proteins with possible involvement in defence responses

The proteins associated with the GO terms “jasmonic acid and ethylene-dependent systemic resistance, ethylene mediated signaling pathway”, “immune response”, “defence response to fungus, incompatible interaction”, “defence response, incompatible interaction”, “chitin binding” and “cell death” were examined in more detail in an attempt to understand the putative mechanisms that could underlie increased resistance in TAG5. Many of these proteins were also associated with other GO terms. These genes, as well as other defence-related genes identified in the data, are listed in Table 1.

**Figure 4:**
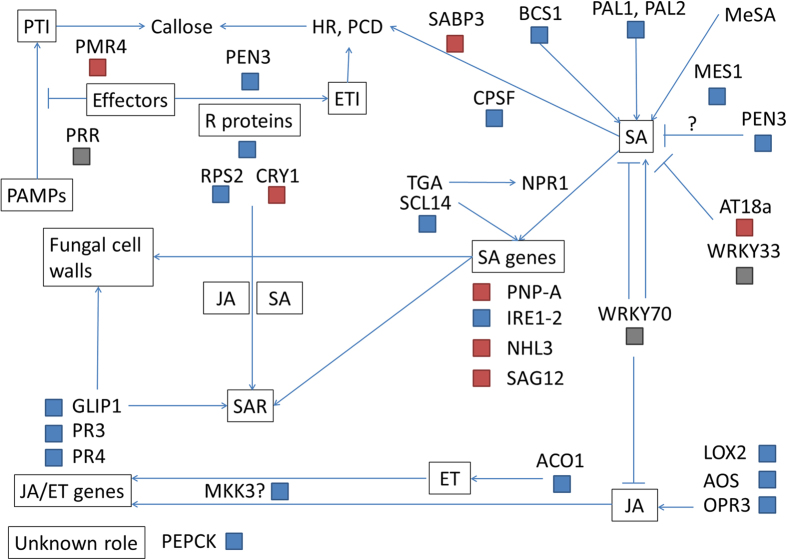
A simplified diagram of the defence signalling components identified in the proteomics data in the inoculated groups (Inoculated-Protein). The pathways are based on previous functional studies. Abbreviations are listed in Table 1. For each gene, the differential abundance is indicated by coloured blocks. Red indicates down-regulation and blue indicates up-regulation.

Several proteins involved in SA signalling and associated processes such as systemic responses to infection, cell death and the hypersensitive response (Fig. 4) were DE in response to inoculation. The Shikimate pathway is involved in many processes, including SA synthesis. Phenylalanine ammonia lyases (PALs) are also important for SAR and pathogen resistance. Several PAL orthologues were DE in the TAG5 proteome in response to wounding and inoculation. SCL14, an orthologue of which was up-regulated after inoculation, interacts with TGA transcription factors and mediates expression of SA-induced genes independently of NPR1^11^. The AtATG18a protein is involved in defence through autophagy and the negative regulation of SA-mediated responses^12^, along with JA and WRKY33^13^. An orthologue of this gene was down-regulated upon inoculation.

The expression of putative systemic acquired resistance (SAR) and induced systemic resistance (ISR) components requiring both SA and JA/ET dependent signalling was altered, such as MES1, which is required for SAR activation^14^,^15^,^16^. The NBS-LRR protein RPS2 has been studied extensively and has a role in SAR^17^,^18^. It was recently found that JA is also involved in promoting the ETI response mediated by RPS2^19^. CRY1 is a positive regulator of SAR and PR gene expression as well as R protein mediated resistance^20^. GLIP1 induces systemic resistance, possibly targets fungal cell walls^21^,^22^, and appears to be an important regulator of ET-mediated defence against necrotrophs^23^. MKK3 is involved in JA/ET-mediated ISR^24^. All of these proteins were up-regulated in the TAG5 inoculated proteome, except CRY1, which was down-regulated.

Based on previous microscopy results^7^ suggesting that *C. austroafricana* spreads via cell wall pits, callose deposition could be important for limiting pathogen spread. Several proteins with putative involvement in callose formation were down-regulated in TAG5 upon inoculation while an orthologue of PEN3, which is required for callose deposition and disease resistance in *Arabidopsis*^25^, was up-regulated (Fig. 4).

Histochemical analyses showed that cell damage and death occur at the site of inoculation, but are limited after wounding. This is consistent with the DE of proteins with putative roles in cell death only after inoculation of TAG5. Orthologues of negative regulators of SA-related PCD (AT4G24290) and of apoptosis (AT5G18400) were up-regulated, while some cell death-promoting proteins were also up-regulated (CPSF, FBR11)^26^. An orthologue of barley mlo (MLO1) was also down-regulated after inoculation. Mutants of the *mlo* gene have increased resistance to powdery mildew and the gene has a role in modulating cell death^27^,^28^,^29^. Reduced expression of an apple *mlo* gene also resulted in increased resistance to the powdery mildew pathogen *Podosphaera leucotricha*^30^. A xylem cysteine peptidase 1 (XCP1) orthologue was down-regulated after inoculation. This protein is a possible target of the virulence factor Avr2 of *Cladosporium fulvum*, which inhibits it and other cysteine proteases^31^. However, XCP2 is associated with susceptibility to *Ralstonia solanacearum*^32^. PEN proteins are involved in many processes, including the HR. Mutants show uncontrolled cell death^33^ and higher levels of SA^34^. The presence of a PEN3 orthologue in the up-regulated dataset of TAG5 suggests that SA-mediated cell death is being modulated.

Phospholipase D delta is associated with resistance to *Blumeria graminis,* preventing cell wall penetration and possibly promoting early defence signalling^35^. This protein, along with glyceraldehyde-3-phosphate dehydrogenases, also has a role in H_2_O_2_ signalling^36^. Orthologues of this protein were down-regulated in response to wounding and inoculation of *E. grandis*. This could be due to its role in the early stages of infection which may no longer be required at 3 dpi. Other putative defence proteins were also DE, including putative thaumatins and additional PR family proteins. Collectively, the information from quantitative proteomics is consistent with an important role for SA signalling in TAG5 resistance and with the histochemical observations.

## Discussion

The purpose of this study was to investigate the defence responses of *E. grandis* to *C. austroafricana.* This was achieved by examining the histological changes in the host after inoculation as well as quantitative proteomics.

We found no evidence of drastic anatomical differences between the clones that could affect pathogen spread. While the wounding and inoculation responses were similar in the two clones, histochemical responses unique to inoculation included cell death. This is consistent with differential expression of proteins with expected involvement in cell death only in the inoculated TAG5 samples. Future experiments will focus on quantifying the rate of cell death to confirm whether it differs between the clones. The abundance and composition of gum-like substances formed after wounding and inoculation could also differ between the clones. These histological changes could constitute a physical impediment to pathogen spread and their contribution cannot be excluded based on the current evidence. Using additional histological stains such as ruthenium red (which stains pectins and gums), the main constituents of this material can be determined, which will guide the selection of secondary metabolites for quantitative analysis. The presence of several DE proteins associated with cell death could indicate the occurrence of a hypersensitive response. Histochemistry of unfixed tissue with 3,3′-diaminobenzidine tetrahydrochloride (DAB) could reveal whether hydrogen peroxide, which is associated with the HR, increases in abundance during infection.

The current definitions of fungal lifestyles state that biotrophs derive nutrients from living cells, while necrotrophs feed on killed tissue and hemibiotrophs use a combination of these strategies^37^,^38^. Certain host responses also tend to be effective against each type of pathogen, although this is not always the case. Responses to necrotrophs would typically involve JA and ET signalling, while SA-mediated responses are associated with resistance to biotrophs and hemibiotrophs^39^,^40^,^41^. Information about feeding strategy and, in some cases, effective host responses can contribute to the identification of pathogen lifestyle^37^. These definitions are not always clear and they are likely to evolve as new interactions are studied. This can complicate the classification of a pathogen according to lifestyle. Previous studies on *E. grandis* and *C. austroafricana* revealed an important role for SA in increased antifungal resistance^3^,^5^. It was also recently shown that *C. austroafricana* may occur as an endophyte in *Syzygium* species^42^, suggesting that this fungus can survive without causing extensive host cell damage. In this study, fungal hyphae were observed in living and dead cells of both clones, suggesting the possibility of a hemibiotrophic interaction in this pathosystem. However, additional information, such as whether specialised feeding structures form during infection, will be required to determine pathogen lifestyle with more certainty. This could be resolved with scanning electron microscopy.

Consistent with previous transcriptomics and hormone profiling experiments, the proteomics analysis of the TAG5 responses to inoculation suggests a central role for SA signalling for defence against *C. austroafricana*. Responses unique to inoculation suggested the involvement of processes typically associated with SA-mediated processes, including cell death, R protein induction and systemic resistance. Certain defence-related genes were expressed at the RNA and protein levels, providing multiple lines of evidence for their involvement in the interaction.

This model pathosystem has been used to study several aspects of the interaction between *C. austroafricana* and *E. grandis*. The location and spread of the pathogen as well as putative fungal pathogenicity factors expressed during infection have been described^7^. Phytohormone treatment and gene expression profiling experiments revealed antagonism between SA and JA as well as the importance of SA for resistance^5^. Transcriptomics and phytohormone profiling experiments led to the identification of several candidate defence genes and revealed the complexity of the interaction^3^. The results presented here contribute to this knowledge by adding information on the histochemical changes in the host during infection, supporting the roles of several candidate defence genes including several identified with transcriptomics (Table 1), and by distinguishing between host responses to wounding and inoculation. The current knowledge about this model pathosystem can be used to guide future functional studies aiming to identify the molecular determinants of increased resistance of *E. grandis* to the fungal pathogen *C. austroafricana*.

## Methods

### Inoculation and sampling

Ramets of the *E. grandis* clones TAG5 (moderately resistant) and ZG14 (highly susceptible) obtained from Mondi were inoculated as described previously^3^,^5^,^7^. The plants were 2–3 years old with a stem diameter of approximately 1 cm. The *C. austroafricana* isolate CMW 2113 (Forestry and Agricultural Biotechnology Institute culture collection; Centraalbureau voor Schimmelcultures KNAW Fungal Biodiversity Center–CBS 112916, Agricultural Research Council National Collection of Fungi–PREM 58023, dried culture) was cultured on 2% malt extract agar (MEA). Stems were inoculated approximately 30 cm above the base using a 5 mm diameter cork borer to remove the bark. A cork borer was used to cut pieces of sterile agar (for wounded controls) or agar covered in mycelium (for inoculated samples) and these pieces were placed inside the artificial wound with mycelium towards the xylem (in inoculated samples). The excised bark was carefully placed on top of the agar and the wound sealed with Parafilm M (Bemis Company) to prevent desiccation. For light microscopy, the stems were cut around the wounding or inoculation site and dissected for tangential longitudinal and cross sectioning. The excised stem pieces were immediately fixed in FAA (5% formalin, 5% acetic acid, 45% ethanol). Stem pieces of wounded and inoculated plants were placed on 2% malt extract agar to confirm the presence of *C. austroafricana* by reisolation. Two separate inoculation trials were performed for the microscopy and proteomics analyses. Lesions were measured at 3, 7, 14, 21 and 42 dpi with at least three biological replicates per treatment at each time point. At least two different plants were observed at each time point for the histochemical analyses of host responses.

For proteomics analysis, the wounded control and inoculated groups each contained three biological replicates; each of these biological replicates contained material from four individual ramets. For each ramet, a section of stem 50 mm in length was excised from around the central inoculation site. The unwounded control group contained two biological replicates, each consisting of a section of stem 80 mm in length (ranging from approximately 26–34 cm from the base of the stem). A total of 3–5 g tissue was harvested for proteomics analysis per replicate. The bark was removed, each sample was split longitudinally into 2–4 pieces to facilitate homogenisation, flash frozen in liquid nitrogen and stored at −80 °C.

### Protein extraction

A phenol-based extraction method was optimised based on previous approaches to protein extraction from recalcitrant material^43^,^44^,^45^. Briefly, the tissue of each biological replicate was homogenised into a fine powder (for approximately 1–2 minutes) using an electric grinder pre-cooled with liquid nitrogen, taking care to prevent thawing of the material. The homogenised tissue was resuspended by vortexing in 3–5 mL of extraction buffer (0.7 M sucrose, 0.5 M EDTA, 0.1 M Tris base, 0.1 M KCl, 1% polyvinylpolypyrrolidone, 1% (w/v) Triton X-100, 1 mM phenylmethylsulfonyl fluoride and 2% 2-mercaptoethanol, pH 8, cooled to 4 °C) per gram of tissue. An equal volume of room temperature UltraPure Buffer-Saturated Phenol (Life Technologies, pH 8) was added to the homogenate and the mixture was vortexed thoroughly. The phenol and aqueous phases were separated by centrifugation at 30 000 × g and 4 °C for 30 minutes in a Beckman ultracentrifuge (SW28 rotor) using thin-walled polypropylene Beckman tubes (326823, Beckman Coulter). Beckman tubes and Eppendorf Lobind microcentrifuge tubes (Sigma-Aldrich) with low binding affinity for proteins were used to limit protein loss. High speed centrifugation further increased protein recovery. The phenol phase was carefully removed, four to five volumes of −20 °C precipitation solution (0.1 M ammonium acetate in methanol) were added to the phenol phase, the tube was inverted to mix the phases, and the solution incubated overnight at −20 °C to precipitate proteins. The solution was centrifuged at 30 000 × g and 4 °C for 15 minutes to collect precipitated proteins. The supernatant was discarded carefully and the pellet washed in −20 °C methanol, incubated at −20 °C for two hours, and centrifuged at 30 000 × g for 15 min at 4 °C. The pellet was subsequently washed in the same way using −20 °C acetone containing 0.1% ME and twice in −20 °C acetone. The acetone was discarded and pellets were resuspended in 1.4 mL clean acetone and transferred to Eppendorf Lobind tubes for storage.

Protein quality was assessed with SDS-PAGE using a 12% polyacrylamide gel at 110 V and 250 mA and Coomassie Brilliant Blue staining. Total protein quantity was estimated with a Pierce Coomassie (Bradford) Assay kit (Thermo Scientific, catalogue number 23200) according to the manufacturer’s instructions, using bovine serum albumin (BSA) as a standard.

The samples were shipped to Beijing Genomics Institute (BGI) at −70 °C. The samples were centrifuged at 30 000 × g and 4 °C for 15 minutes, whereafter the pellets were air dried and resuspended in 300 μL of lysis buffer each (7 M urea, 2 M thiourea, 4% Nonidet P-40 and 20 mM Tris, pH 8–8.5). The suspension was sonicated at 200 W for 15 minutes and centrifuged at 30 000 × g and 4 °C for 15 minutes. The supernatant was removed and treated with 10 mM DTT (dithiothreitol) for 60 minutes at 56 °C to reduce disulfide bonds. Cysteine residues were blocked (alkylated) with 55 mM IAM (iodoacetamide) for 45 minutes in the dark. The samples were centrifuged at 30 000 × g and 4 °C for 15 minutes and the supernatant was retained for subsequent analyses. BGI also assessed protein quantity with a Bradford assay and protein integrity with SDS-PAGE.

### iTRAQ experiment

After verifying the protein quantity and quality, a total of 100 μg protein from each sample was digested for 4 hours at 37 °C using TrypsinGold (protein:trypsin ratio of 20:1). The digestion was repeated for 8 hours. The peptides were vacuum centrifuged, dissolved in 0.5 M TEAB, and labelled according to the iTRAQ Reagent 8-Plex Kit protocol (AB Sciex Inc., MA, USA). The two unwounded controls were labelled with iTRAQ reagents 113 (1A) and 116 (1B), the three wounded controls with 114 (2A), 117 (2B) and 119 (2C), and the three inoculated samples with 115 (3A), 118 (3B) and 121 (3C). The samples were separated into 20 fractions by charge using strong cation exchange chromatography. Digested peptides were dissolved in buffer A (5% acetonitrile, pH 9.8), loaded onto a Gemini C18 column (Phenomenex) and eluted at a flow rate of 1 mL/min with a gradient of 5% buffer B (95% ACN, pH 9.8) for 10 min, 5–35% buffer B for 40 min, 35–95% buffer B for 1 min, 95% buffer B for 3 min and decreasing to 5% within 1 min before equilibration with 5% buffer B for 10 min. Fractions were collected at 1 minute intervals and the final fractions were vacuum dried.

HPLC MS/MS analysis of the samples was performed by BGI as described previously^4^, except that solvent B had a composition of 98% acetonitrile, 0.1% formic acid and that a gradient of 5–35% buffer D (98% acetonitrile, 0.1% formic acid) was used and maintained for 5 minutes rather than 4 minutes. Instead of returning to solvent C for 1 minute, the elution concluded with 5% buffer D for one minute. The data-dependent procedure was applied to the fifteen most abundant precursor ions.

### Data analysis

The data analysis was performed by BGI using Mascot 2.3.02 combined with iQuant^46^. The *Eucalyptus grandis* (genome version 2.0, Phytozome) and *Chrysoporthe austroafricana* (Genbank JYIP00000000)^47^ predicted proteomes were combined in a concatenated protein database for the spectrum-database search (Table 2). The design of the quantitative proteomics experiment allowed the identification of responses to wounding as well as inoculation. The following comparisons were made: Wounded/Unwounded, Inoculated/Unwounded, and Inoculated/Wounded. For the differentially expressed proteins, a p-value of less than 0.05 and a fold change above 1.2 were required. For the target-decoy search, a false discovery rate below 1 was required.

Significantly different proteins were analysed with MapMan version 3.5.1R2^48^ and BiNGO 3.0.3 in Cytoscape v.3.4.0^49^ (Benjamini-Hochberg false discovery rate (FDR) correction and a p-value < 0.05) to identify pathways and processes that could be involved in the interaction. For MapMan analysis, the *E. grandis* mapping was used. The annotation and ontology files (*Arabidopsis thaliana*) for BiNGO were downloaded from www.geneontology.org on 19 July 2016.

In a previous study^3^, RNA-Sequencing and RT-qPCR validation of selected genes were performed with stem tissue of *E. grandis* inoculated with *C. austroafricana* (CMW 2113) harvested at 3 dpi. Lists of differentially expressed genes that were identified in this study were updated with the latest *E. grandis* annotation (v2.0) and newer ontology and annotation files in BiNGO. These data were used to validate the proteomics results reported here.

### Light microscopy

Samples were prepared for light microscopy as described previously^7^. Fixed excised stem pieces were sectioned with a sliding microtome, or dehydrated with a butanol series, embedded in paraffin wax (Sigma-Aldrich, 76242), mounted on wooden blocks and sectioned with a rotary microtome^7^. Both cross-sections and tangential longitudinal sections at the inoculation site were examined. These sections were stored on glass microscope slides and the wax removed with 100% xylene prior to staining. Sections were stained with Safranin O (uniLAB) and Fast Green FCF (uniLAB), and mounted in Entellan (Merck Millipore). Fixed sections were mounted directly in Lugol’s solution (1 I:2 KI (w/v)) for detecting starch. Fresh sliding microtome sections were mounted in neutral red solution (0.85 M potassium nitrate, 0.01% (w/v) neutral red, in 0.02 M phosphate buffer, pH 7.5) according to the method for vital staining described by Woods *et al*.^9^, with the exception that no vacuum was applied to the samples. Helicon Focus (HeliconSoft) was used to compile images from multiple focal planes when required.

## Additional Information

**How to cite this article:** Zwart, L. *et al*. Evidence for salicylic acid signalling and histological changes in the defence response of *Eucalyptus grandis* to *Chrysoporthe austroafricana. Sci. Rep.* **7**, 45402; doi: 10.1038/srep45402 (2017).

**Publisher's note:** Springer Nature remains neutral with regard to jurisdictional claims in published maps and institutional affiliations.

## Supplementary Material

Supplementary Information

## Figures and Tables

**Table 1:** Defence-related GO terms and their associated *A. thaliana* and *E. grandis* genes.

TAIR10 ID	Name	Description	*E. grandis* ID	Protein Inoculated
**SA**				
AT2G37040	PAL1	Phenylalanine ammonia lyase 1	Eucgr.G02848	**Up**
AT3G53260	PAL2	Phenylalanine ammonia lyase 2	*Eucgr.J00907*	**Up**
			*Eucgr.J01079*	Up
AT1G30460	CPSF30	cleavage and polyadenylation specificity factor 30	Eucgr.E02496	Up
AT1G59870	ABCG36, PDR8, PEN3	ABC-2 and Plant PDR ABC-type transporter family protein	*Eucgr.F03072*	Up
AT3G50930	BCS1	cytochrome BC1 synthesis	Eucgr.J02373	**Up**
AT2G17520	IRE1-2, IRE1A	Endoribonuclease/protein kinase IRE1-like	Eucgr.J02544	Up
AT4G37870	PCK1, PEPCK	phosphoenolpyruvate carboxykinase 1	*Eucgr.I00628*	**Up**
AT5G06320	NHL3	NDR1/HIN1-like 3	Eucgr.A02597	**Down**
AT2G18660	PNP-A	Plant natriuretic peptide A	Eucgr.C01794	Down
AT5G45890	SAG12	senescence-associated gene 12	Eucgr.D00496	Down
			Eucgr.D00500	Down
			Eucgr.D00502	Down
			Eucgr.L00918	Up
**SAR**				
AT1G07530	GRAS2, SCL14	SCARECROW-like 14	*Eucgr.B02337*	Up
AT2G23620	MES1	methyl esterase 1	Eucgr.I01002	Up
			*Eucgr.I01005*	Up
AT4G26090	RPS2	NB-ARC domain-containing disease resistance protein	Eucgr.G00412	Up
			Eucgr.G00714	Up
			Eucgr.L00073	Up
AT5G40440	MKK3	mitogen-activated protein kinase kinase 3	Eucgr.I01749	Up
AT4G08920	BLU1, CRY1	cryptochrome 1	*Eucgr.F00326*	**Down**
AT5G40990	GLIP1	GDSL lipase 1	Eucgr.I02146	**Up**
**ET/JA**				
AT3G62770	AtATG18a	Transducin/WD40 repeat-like superfamily protein	*Eucgr.B01921*	Down
AT4G11650	OSM34	osmotin 34	*Eucgr.D01888*	Up
			Eucgr.H03863	Up
AT3G12500	CHI-B, PR-3, PR3	basic chitinase	*Eucgr.I01495*	Up
			*Eucgr.J02519*	Up
AT3G04720	HEL, PR-4, PR4	pathogenesis-related 4	Eucgr.H04329	Up
AT3G45140	LOX2	Lipoxygenase 2	Eucgr.J00819	Up
			Eucgr.L01891	**Up**
**HR/PCD**				
AT3G54420	ATEP3, CHIV, EP3	homolog of carrot EP3-3 chitinase	Eucgr.A00021	**Up**
			Eucgr.K02166	**Up**
AT4G24290	NA	MAC/Perforin domain-containing protein	Eucgr.I00007	**Up**
AT4G37930	SHM1, SHMT1	serine transhydroxymethyltransferase 1	Eucgr.I00580	**Down**
AT3G01500	CA1, SABP3	carbonic anhydrase 1	Eucgr.I01790	**Down**
AT4G35350	XCP1	xylem cysteine peptidase 1	Eucgr.F03589	Down
AT4G35790	PLDDELTA	phospholipase D delta	Eucgr.I02299	Down
			Eucgr.I02300	**Down**
AT4G36480	FBR11, LCB1	long-chain base1	Eucgr.D00310	Up
AT5G12080	MSL10	mechanosensitive channel of small conductance-like 10	Eucgr.C00712	Down
AT5G18400	NA	Cytokine-induced anti-apoptosis inhibitor 1, Fe-S biogenesis	Eucgr.J00852	Up
AT4G02600	MLO1	Seven transmembrane MLO family protein	Eucgr.D01126	Down
AT4G35090	CAT2	catalase 2	Eucgr.F01776	Down
			Eucgr.F03557	**Down**
**Other**				
ATCG00480	NA	ATP synthase subunit beta	Eucgr.F02916	**Down**
			Eucgr.H03409	**Down**
			Eucgr.J02738	**Down**
AT4G03550	GSL05, GSL5, PMR4	glucan synthase-like 5	Eucgr.K01027	Down
AT4G04970	GSL01, GSL1	glucan synthase-like 1	Eucgr.D00621	Down

Differentially expressed proteins identified with iTRAQ are listed. Proteins present in both the Wounded/Unwounded and Inoculated/Unwounded comparisons are shown in bold type. Genes that share DE patterns in the proteomics data and previous transcriptomics data are italicised. The “Protein-Inoculated” group includes information from inoculated vs. wounded and inoculated vs. unwounded. Additional information about the proteins is shown in Supplementary Table S1.

**Table 2:** Parameters for spectrum-database search.

Parameter (Mascot)	Value
Type of search	MS/MS ion search
Enzyme	Trypsin
Fragment mass tolerance	0.05 Da
Mass values	Monoisotopic
Charge	+2, +3, +4
Missed cleavages	2
Decoy (0 = off, 1 = on)	1
Variable modifications	Oxidation (M), iTRAQ8plex (Y)
Fixed modifications	Carbamidomethyl (C), iTRAQ8plex (N-term), iTRAQ8plex (K)
**Parameter (iQuant)**	**Value**
Quant_peptide	Use All Unique Peptides
Quant_number	At least one unique spectrum
Normalisation	Variance stabilisation normalisation (VSN)
Protein_Ratio	Weighted average
Statistical analysis	Permutation Tests

## References

[b1] Myburg A. A. . The genome of *Eucalyptus grandis*. Nature 510, 356–362 (2014). 24919147 10.1038/nature13308

[b2] Christie N., Tobias P. A., Naidoo S. & Külheim C. The *Eucalyptus grandis* NBS-LRR gene family: physical clustering and expression hotspots. Front. Plant Sci. 6, 1–16 (2016). 10.3389/fpls.2015.01238 PMC4709456 26793216

[b3] Mangwanda R., Myburg A. A. & Naidoo S. Transcriptome and hormone profiling reveals *Eucalyptus grandis* defence responses against *Chrysoporthe austroafricana*. BMC Genomics 16, 319 (2015). 25903559 10.1186/s12864-015-1529-x PMC4405875

[b4] Chen Q. . Transcriptome and proteome analysis of *Eucalyptus* infected with *Calonectria pseudoreteaudii*. J. Proteomics 115, 117–131 (2015). 25540935 10.1016/j.jprot.2014.12.008

[b5] Naidoo R., Ferreira L., Berger D. K., Myburg A. A. & Naidoo S. The identification and differential expression of *Eucalyptus grandis* pathogenesis-related genes in response to salicylic acid and methyl jasmonate. Front. Plant Sci. 4, 43 (2013). 23508356 10.3389/fpls.2013.00043 PMC3589731

[b6] van Heerden S. W., Amerson H. V., Preisig O., Wingfield B. D. & Wingfield M. J. Relative pathogenicity of *Cryphonectria cubensis* on *Eucalyptus* clones differing in their resistance to *C. cubensis*. Plant Dis. 89, 659–662 (2005). 10.1094/PD-89-0659 30795393

[b7] Mangwanda R. . Localization and transcriptional responses of *Chrysoporthe austroafricana* in *Eucalyptus grandis* identify putative pathogenicity factors. Frontiers in Microbiology 7, 1953 (2016). 28008326 10.3389/fmicb.2016.01953 PMC5143476

[b8] Horbach R., Navarro-Quesada A. R., Knogge W. & Deising H. B. When and how to kill a plant cell: infection strategies of plant pathogenic fungi. J. Plant Physiol. 168, 51–62 (2011). 20674079 10.1016/j.jplph.2010.06.014

[b9] Woods A. M., Fagg J. & Mansfield J. W. Fungal development and irreversible membrane damage in cells of *Lactuca sativa* undergoing the hypersensitive reaction to the downy mildew fungus *Bremia lactucae*. Physiol. Mol. Plant Pathol. 32, 483–497 (1988).

[b10] Lee M., Qi M. & Yang Y. A Novel Jasmonic Acid-Inducible Rice myb Gene Associates with Fungal Infection and Host Cell Death. Mol. Plant Microbe Interact. 14, 527–535 (2001). 11310740 10.1094/MPMI.2001.14.4.527

[b11] Fode B., Siemsen T., Thurow C., Weigel R. & Gatz C. The *Arabidopsis* GRAS protein SCL14 interacts with class II TGA transcription factors and is essential for the activation of stress-inducible promoters. Plant Cell 20, 3122–3135 (2008). 18984675 10.1105/tpc.108.058974 PMC2613660

[b12] Lenz H. D. . Autophagy differentially controls plant basal immunity to biotrophic and necrotrophic pathogens. Plant J. 66, 818–830 (2011). 21332848 10.1111/j.1365-313X.2011.04546.x

[b13] Lai Z., Wang F., Zheng Z., Fan B. & Chen Z. A critical role of autophagy in plant resistance to necrotrophic fungal pathogens. Plant J. 66, 953–968 (2011). 21395886 10.1111/j.1365-313X.2011.04553.x

[b14] Park S.-W., Kaimoyo E., Kumar D., Mosher S. & Klessig D. F. Methyl Salicylate Is a Critical Mobile Signal for Plant Systemic Acquired Resistance. Science 318, 113–116 (2007). 17916738 10.1126/science.1147113

[b15] Shah J. Plants under attack: systemic signals in defence. Curr. Opin. Plant Biol. 12, 459–464 (2009). 19608451 10.1016/j.pbi.2009.05.011

[b16] Kumar D. & Klessig D. F. High-affinity salicylic acid-binding protein 2 is required for plant innate immunity and has salicylic acid-stimulated lipase activity. Proc. Natl. Acad. Sci. 100, 16101–16106 (2003). 14673096 10.1073/pnas.0307162100 PMC307699

[b17] Cameron R. K., Paiva N. L., Lamb C. J. & Dixon R. a. Accumulation of salicylic acid and PR-1 gene transcripts in relation to the systemic acquired resistance (SAR) response induced by *Pseudomonas syringae* pv. tomato in Arabidopsis. Physiol. Mol. Plant Pathol. 55, 121–130 (1999).

[b18] Tao Y., Yuan F. H., Leister R. T., Ausubel F. M. & Katagiri F. Mutational analysis of the *Arabidopsis* nucleotide binding site- leucine-rich repeat resistance gene *RPS2*. Plant Cell 12, 2541–2554 (2000). 11148296 10.1105/tpc.12.12.2541 PMC102236

[b19] Liu L. . Salicylic acid receptors activate jasmonic acid signalling through a non-canonical pathway to promote effector-triggered immunity. Nat. Commun. 7, 13099 (2016). 27725643 10.1038/ncomms13099 PMC5062614

[b20] Wu L. & Yang H. Q. CRYPTOCHROME 1 is implicated in promoting R protein-mediated plant resistance to *Pseudomonas syringae* in *Arabidopsis*. Mol. Plant 3, 539–548 (2010). 20053798 10.1093/mp/ssp107

[b21] Kwon S. J. . GDSL lipase-like 1 regulates systemic resistance associated with ethylene signaling in *Arabidopsis*. Plant J. 58, 235–245 (2009). 19077166 10.1111/j.1365-313X.2008.03772.x

[b22] Oh I. S. . Secretome analysis reveals an *Arabidopsis* lipase involved in defense against *Alternaria brassicicola*. Plant Cell 17, 2832–2847 (2005). 16126835 10.1105/tpc.105.034819 PMC1242276

[b23] Kim H. G. . GDSL lipase 1 regulates ethylene signaling and ethylene-associated systemic immunity in *Arabidopsis*. FEBS Lett. 588, 1652–1658 (2014). 24631536 10.1016/j.febslet.2014.02.062

[b24] Takahashi F. . The mitogen-activated protein kinase cascade MKK3-MPK6 is an important part of the jasmonate signal transduction pathway in *Arabidopsis*. Plant Cell 19, 805–818 (2007). 17369371 10.1105/tpc.106.046581 PMC1867372

[b25] Clay N. K., Adio A. M., Denoux C., Jander G. & Ausubel F. M. Glucosinolate Metabolites Required for an *Arabidopsis* Innate Immune Response. Science 323, 95–101 (2009). 19095898 10.1126/science.1164627 PMC2630859

[b26] Shi L. . Involvement of sphingoid bases in mediating reactive oxygen intermediate production and programmed cell death in *Arabidopsis*. Cell Res. 17100, 1030–1040 (2007). 10.1038/cr.2007.100 18059378

[b27] Büschges R. . The barley *Mlo* gene: a novel control element of plant pathogen resistance. Cell 88, 695–705 (1997). 9054509 10.1016/s0092-8674(00)81912-1

[b28] Piffanelli P.. The Barley MLO Modulator of Defense and Cell Death Is Responsive to Biotic and Abiotic Stress Stimuli. Plant Physiol. 129, 1076–1085 (2002). 12114562 10.1104/pp.010954 PMC166502

[b29] Jørgensen I. H. Discovery, characterization and exploitation of Mlo powdery mildew resistance in barley. Euphytica 63, 141–152 (1992).

[b30] Pessina S. . The knock-down of the expression of MdMLO19 reduces susceptibility to powdery mildew (*Podosphaera leucotricha*) in apple (*Malus domestica*). Plant Biotechnol. J. 14, 2033–2044 (2016). 26997489 10.1111/pbi.12562 PMC5043462

[b31] van Esse H. P. . The *Cladosporium fulvum* Virulence Protein Avr2 Inhibits Host Proteases Required for Basal Defense. Plant Cell Online 20, 1948–1963 (2008). 10.1105/tpc.108.059394 PMC2518240 18660430

[b32] Zhang B. . PIRIN2 stabilizes cysteine protease XCP2 and increases susceptibility to the vascular pathogen *Ralstonia solanacearum* in *Arabidopsis*. Plant J. 79, 1009–1019 (2014). 24947605 10.1111/tpj.12602 PMC4321228

[b33] Johansson O. N. . Role of the penetration-resistance genes PEN1, PEN2 and PEN3 in the hypersensitive response and race-specific resistance in *Arabidopsis thaliana*. Plant J. 79, 466–476 (2014). 24889055 10.1111/tpj.12571

[b34] Stein M. . Arabidopsis PEN3/PDR8, an ATP Binding Cassette Transporter, Contributes to Nonhost Resistance to Inappropriate Pathogens That Enter by Direct Penetration. Plant Cell 18, 731–746 (2006). 16473969 10.1105/tpc.105.038372 PMC1383646

[b35] Pinosa F. . *Arabidopsis* phospholipase Ddelta is involved in basal defense and non-host resistance to powdery mildew fungi. Plant Physiol. 163, 896–906 (2013). 23979971 10.1104/pp.113.223503 PMC3793066

[b36] Guo L. . Cytosolic glyceraldehyde-3-phosphate dehydrogenases interact with phospholipase Dδ to transduce hydrogen peroxide signals in the *Arabidopsis* response to stress. Plant Cell 24, 2200–2212 (2012). 22589465 10.1105/tpc.111.094946 PMC3442596

[b37] Oliver R. P. & Ipcho S. V. S. *Arabidopsis* pathology breathes new life into the necrotrophs-vs.-biotrophs classification of fungal pathogens. Mol. Plant Pathol. 5, 347–352 (2004). 20565602 10.1111/j.1364-3703.2004.00228.x

[b38] Perfect S. E. & Green J. R. Infection structures of biotrophic and hemibiotrophic fungal plant pathogens. Mol. Plant Pathol. 2, 101–108 (2001). 20572997 10.1046/j.1364-3703.2001.00055.x

[b39] Denancé N., Sánchez-Vallet A., Goffner D. & Molina A. Disease resistance or growth: the role of plant hormones in balancing immune responses and fitness costs. Front. Plant Sci. 4, 155 (2013). 23745126 10.3389/fpls.2013.00155 PMC3662895

[b40] Glazebrook J. Contrasting mechanisms of defense against biotrophic and necrotrophic pathogens. Annu. Rev. Phytopathol. 43, 205–227 (2005). 16078883 10.1146/annurev.phyto.43.040204.135923

[b41] Tanaka S., Han X. & Kahmann R. Microbial effectors target multiple steps in the salicylic acid production and signaling pathway. Front. Plant Sci. 6, 349 (2015). 26042138 10.3389/fpls.2015.00349 PMC4436567

[b42] Mausse-Sitoe S. N. D., Rodas C. A., Wingfield M. J., Chen S. & Roux J. Endophytic Cryphonectriaceae on native Myrtales: Possible origin of *Chrysoporthe* canker on plantation-grown *Eucalyptus*. Fungal Biol. 120, 827–835 (2016). 27268243 10.1016/j.funbio.2016.03.005

[b43] Saravanan R. & Rose J. A critical evaluation of sample extraction techniques for enhanced proteomic analysis of recalcitrant plant tissues. Proteomics 4, 2522–2532 (2004). 15352226 10.1002/pmic.200300789

[b44] Carpentier S. C. . Preparation of protein extracts from recalcitrant plant tissues: An evaluation of different methods for two-dimensional gel electrophoresis analysis. Proteomics 5, 2497–2507 (2005). 15912556 10.1002/pmic.200401222

[b45] Faurobert M., Pelpoir E. & Chaïb J. Phenol extraction of proteins for proteomic studies of recalcitrant plant tissues. Plant Proteomics Methods Protoc. 9–14 (2007). 10.1385/1-59745-227-0:9 17093297

[b46] Wen B. . IQuant: An automated pipeline for quantitative proteomics based upon isobaric tags. Proteomics 14, 2280–2285 (2014). 25069810 10.1002/pmic.201300361

[b47] Wingfield B. D. . IMA Genome-F 4: Draft genome sequences of Chrysoporthe austroafricana, Diplodia scrobiculata, Fusarium nygamai, Leptographium lundbergii, Limonomyces culmigenus, Stagonosporopsis tanaceti, and Thielaviopsis punctulata. IMA Fungus 6, 233–248 (2015). 26203426 10.5598/imafungus.2015.06.01.15 PMC4500086

[b48] Thimm O. . MAPMAN: A user-driven tool to display genomics data sets onto diagrams of metabolic pathways and other biological processes. Plant J. 37, 914–939 (2004). 14996223 10.1111/j.1365-313x.2004.02016.x

[b49] Maere S., Heymans K. & Kuiper M. Systems biology BiNGO: a Cytoscape plugin to assess overrepresentation of Gene Ontology categories in Biological Networks. Bioinformatics 21, 3448–3449 (2005). 15972284 10.1093/bioinformatics/bti551

