# Supplementary material for: Evidence for salicylic acid signalling and histological changes in the defence response of Eucalyptus grandis to Chrysoporthe austroafricana
Source: Sci Rep. 2017 Mar 28;7:45402. doi: 10.1038/srep45402 (PMC5368643; doi:10.1038/srep45402)
Supplement: Supplementary Information [file srep45402-s1.zip › Supplementary Figure S1 Figure S2.docx]

# Evidence for salicylic acid signalling and histological changes in the defence response of *Eucalyptus grandis* to *Chrysoporthe austroafricana*

Lizahn Zwart^1^, Dave Kenneth Berger^2^, Lucy Novungayo Moleleki^3^, Nicolaas A. van der Merwe^1^, Alexander A. Myburg^1^, Sanushka Naidoo^1*^

^1^Department of Genetics, Forestry and Agricultural Biotechnology Institute (FABI), Genomics Research Institute (GRI), University of Pretoria, Pretoria, South Africa

^2^Department of Plant and Soil Sciences, Forestry and Agricultural Biotechnology Institute (FABI), Genomics Research Institute (GRI), University of Pretoria, Pretoria, South Africa

^3^Department of Microbiology and Plant Pathology, Forestry and Agricultural Biotechnology Institute (FABI), University of Pretoria, Pretoria, South Africa

**Corresponding author**

Sanushka Naidoo

Email: sanushka.naidoo@up.ac.za

Tel: (+27) 12 420 4974

**
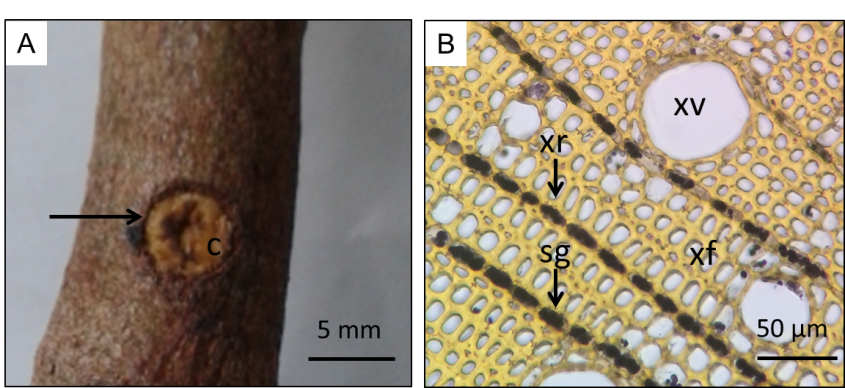
**

**Supplementary Figure S1.** Callus forms at the wound site after artificial wounding of *E. grandis* stems (A). Starch granules are present in xylem ray parenchyma cells after staining cross-sections of *E. grandis* stems with Lugol’s solution (B). c: callus, xv: xylem vessel, xf: xylem fibre, xr: xylem ray, sg: starch granules.


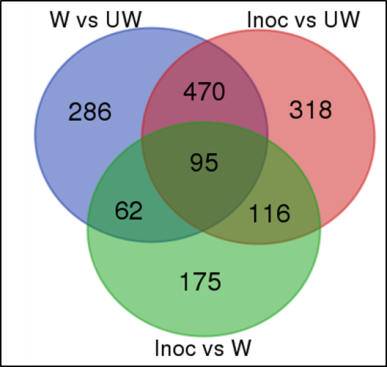


**Supplementary Figure S2.** A Venn diagram comparing the DE proteins (*E. grandis* gene IDs) identified in the Wounded/Unwounded (W vs UW), Inoculated/Unwounded (Inoc vs UW) and Inoculated/Wounded (Inoc vs W) groups. In each case, up- and down-regulated proteins were combined.
